# Indian Ocean Dipole in CMIP5 and CMIP6: characteristics, biases, and links to ENSO

**DOI:** 10.1038/s41598-020-68268-9

**Published:** 2020-07-13

**Authors:** Sebastian McKenna, Agus Santoso, Alexander Sen Gupta, Andréa S. Taschetto, Wenju Cai

**Affiliations:** 1 Australian Research Council (ARC) Centre of Excellence for Climate Extremes and Climate Change Research Centre, The University of New South Wales, Sydney, NSW Australia; 2 Centre for Southern Hemisphere Oceans Research (CSHOR), CSIRO Oceans and Atmosphere, Hobart, TAS Australia; 3 Key Laboratory of Physical Oceanography/Institute for Advanced Ocean Studies, Ocean University of China and Qingdao National Laboratory for Marine Science and Technology, Qingdao, China

**Keywords:** Physical oceanography, Climate and Earth system modelling, Projection and prediction

## Abstract

Accurately representing the Indian Ocean Dipole (IOD) is crucial for reliable climate predictions and future projections. However, El Niño-Southern Oscillation (ENSO) and IOD interact, making it necessary to evaluate ENSO and IOD simultaneously. Using the historical simulation from 32 fifth phase of Coupled Model Intercomparison Project (CMIP5) models and 34 CMIP6 models, here we find that there are some modest changes in the basic characteristics of the IOD and ENSO from CMIP5 to CMIP6. Firstly, there is a slight shift in the seasonality of IOD toward an earlier peak in September in CMIP6, from November in CMIP5. Secondly, inter-model spread in the frequency of ENSO and the IOD has reduced in CMIP6 relative to CMIP5. ENSO asymmetry is still underestimated in CMIP6, based on the skewness of the Niño3 index, while the IOD skewness has degraded from CMIP5. Finally, mean state SST biases impact on the strength of the IOD; the Pacific cold tongue mean state is important in CMIP5, but in CMIP6 the Pacific warm pool mean state is more important.

## Introduction

The Indo-Pacific Oceans host some of the world’s major climate phenomena: the El Niño-Southern Oscillation (ENSO) sourced in the Pacific Ocean^1^, and the Indian Ocean Dipole (IOD)—an ‘ENSO-like’ phenomenon in the Indian Ocean^2^. ENSO is characterised by anomalous warming (El Niño) or cooling (La Niña) over the equatorial Pacific Ocean that peaks around December. The IOD peaks around September–November, with its positive phase (pIOD) characterised by anomalously cool sea surface off Java and Sumatra, and anomalous warming in the tropical western Indian Ocean; vice versa for the negative phase (nIOD). Both ENSO and the IOD develop through a positive Bjerknes feedback^3^ whereby SST zonal gradient, zonal wind stress, thermocline depth and upwelling interact to amplify perturbation from the background state. The two climate drivers are linked through the Walker Circulation that connects the Pacific and Indian Oceans, such that El Niño often occurs with a pIOD, and La Niña with an nIOD. Co-occurrences of pIOD with La Niña, and nIOD with El Niño are rare in observations^4,5^. Given their pronounced impact on global and regional climate, accurate representation of ENSO and IOD in climate models is crucial to produce reliable climate forecasts and future projections. In this regard, monitoring progress has been facilitated through the Climate Model Intercomparison Project (CMIP) which has pointed out both improvements and challenges in simulating ENSO and IOD^6–9^. As results from the sixth phase of CMIP (CMIP6) are now available, it is of interest to re-evaluate our progress in modelling the IOD, ENSO, and their link.

ENSO has been a subject of intense research for many decades, understandably due to its dominant influence on global climate variability^10^. The IOD also has pronounced impact on a global scale^11^, particularly on Indian Ocean-rim countries that host billions in human population. Some impacts of the IOD include variability of the Indian monsoon, with positive events associated with higher rainfall^12,13^. East African rainfall^2^ and African short rains are impacted, with pIODs increasing rainfall^14,15^. The IOD affects rainfall and temperature across central to southern Australia^16^, with some of south-eastern Australia’s worst droughts linked to a long-term lack of nIOD events^17^ and some pIOD events contributing to bushfires^18^.

ENSO is connected to the Indian Ocean via an atmospheric bridge and can trigger an IOD event and modulate its evolution^19–21^. Recent research has indicated that the IOD is a combination of internally forced and ENSO driven phenomenon^20,22^. Two of the strongest pIOD events in recent history have occurred with and without El Niño being present; the 1997 pIOD co-occurred with El Niño, and the 2019 pIOD occurred independently. Due to background seasonal changes in the IO basin, the IOD is seasonally phase-locked to begin in June to August (JJA), peak in September to November (SON), and decay rapidly in December to February (DJF). However, research has shown that IOD onset, peak, and decay timing can vary to some extent due to ENSO^19–21^. Some studies have suggested that like ENSO, which exhibits different flavours of events^5,23^, IOD also appears to exhibit event diversity. For instance, IOD has been classified into three different event types based on their timing and the influence of ENSO^20,22,24^: (i) internally forced IOD events peaking in SON, (ii) early-onset IOD unrelated to ENSO events, typically peaking in JJA (before the IOD usual peak season in SON)^20^; and, (iii) those triggered by ENSO^20,22^ that typically last longer than an independent IOD event. The unseasonable IOD type (ii) has been proposed to be IODs aborted by downwelling warm Kelvin waves, excited by intraseasonal disturbances^25^. Additionally, spatial diversity in the IOD is shown to exist, with an ‘IOD Modoki’ characterised by SST anomalies in the central IO^26^. This suggests that the IOD has a complex behaviour that results from a combination of internal processes in the Indian Ocean and remote influences such as ENSO, potentially presenting further challenges in modelling the IOD. In this study however, we focus on IOD diversity only in terms of its co-occurrence with ENSO, that is, examining ENSO-independent and ENSO co-occurring IOD events.

An accurate representation of IOD flavours in climate models has shown to be challenging^27^. Climate models suffer from biases that affect the way IOD is simulated, typically influencing the representation of IOD strength and diversity^8,28,29^. An overly strong IOD amplitude has been a stubborn bias through generations of climate models associated with biases in SST, thermocline and wind feedbacks^30^. Overly strong climatological easterly wind in the east IO leads to overly shallow thermocline, favouring stronger positive Bjerknes feedback that gives rise to stronger SST anomalies during IOD events. These model biases may also affect future projections in ways that are not fully understood^28,31^.

In addition to models exhibiting biases affecting the IOD, model biases also plague the representation of ENSO and its teleconnections^32–34^. The Pacific Ocean cold tongue extends too far west in many CMIP5 models, and there is also a double Inter-Tropical Convergence Zone (ITCZ) problem in many models^35^. This also impacts on model ability to reproduce ENSO diversity, with many CMIP5 models not able to reproduce the observed diversity of ENSO events (warm pool and cold tongue events)^36,37^.

This study aims to examine the characteristics of IOD in CMIP5 and CMIP6 models, its interactions with ENSO, and possible impact of mean state biases. Our focus is on the general properties and measures of IOD and ENSO. We document changes on the representation of IOD and ENSO going from CMIP5 to CMIP6. The rest of the paper is organised as follows. The models, observational data, and analysis methods are outlined in section “Methods”. Results are presented in section “Results”, describing the basic properties of the IOD and ENSO, such as seasonality, amplitude and frequency, as well as the relationships between ENSO and IOD, and link with mean state biases. Section “Discussion and conclusions” concludes the paper with a summary and discussions.

## Methods

We examine monthly SST outputs from 32 CMIP5 and 34 CMIP6 historical simulations (models listed in Supplementary Tables S1 and S2). Only one ensemble member for each model is used, i.e., r1i1p1 integration for CMIP5, and mostly r1i1p1f1, with select models using variant f2 for CMIP6. The observational product we use for SST is the Hadley Centre Sea Ice and Sea Surface Temperature (HadISST)^38^ dataset for all comparisons, and NOAA Extended Reconstructed Sea Surface Temperature Version 5 (ERSSTv5) to add confidence in results^39^. We take the 1950–2018 period as the reference base in observations to better understand the modern period of the IOD—and also to have a robust sample of observations.

For ease of analysis the output of each model is remapped onto a 1° × 1° regular grid, similar to previous studies^30^. We use the whole timeseries (1850–2005) for each model, to get a robust representation of variability, with diversity in events. To remove the impact of decadal variability, and any warming signal present, we remove a 20-year running mean from each timeseries, effectively shortening the timeseries by 10 years on either end. It should be kept in mind the different period and length of observation to compare the model results against. The observation is not meant to be a strict reference but to serve as a guide to facilitate model intercomparison.

Climatic phenomena are quantified using different indexes, based on SST anomalies (SSTA). To quantify the IOD, we used the Dipole Mode Index (DMI), defined as the SSTA difference between the West Tropical Indian Ocean (WTIO), and South East Tropical Indian Ocean (SETIO)^2^. The WTIO region is 50°E–70°E, 10°S–10°N, and the SETIO region is 90°E–110°E, 10°S–0°N. ENSO has a variety of different indexes measuring its strength and evolution. Here we use the Niño3, Niño3.4, and Niño4 SST indexes^40^. These indexes are defined as SST averaged over the following regions: Niño3 (5°N–5°S, 150°W–90°W), Niño3.4 (5°N–5°S, 170°W–120°W), Niño4 (5°N–5°S, 160°E–150°W). The Niño3.4 index is used to describe the evolution and strength of ENSO events in general. As the Niño3.4 region lies between Niño3 and Niño4 regions, it captures both eastern Pacific and Central Pacific flavour of events.

### Event classification

We classify an IOD as when the mean SON DMI exceeds one standard deviation. Similarly, ENSO events are defined as when the DJF Niño3.4 index exceeds its one standard deviation. In examining the occurrences of IOD and ENSO events, we consider a two-year progression. The IOD evolves in year (0) and decays by the end of year (0). ENSO evolves over year (0) to peak in D(0)JF(1), and decay into year (1). To determine a metric of the influence of ENSO on the IOD, we looked at when IOD events occur in different seasons—JJA, JJASON, SON (these seasons are mutually exclusive—events are not double counted). For each of these seasons we classified an IOD as when the season (e.g., JJA) DMI exceeds the seasonal standard deviation mean (e.g., JJA), similar to classifying an IOD above. We also consider individual IOD events not co-occurring with ENSO (hereafter referred to as “pure IOD”) and ENSO co-occurring IODs. For simplicity, a pure IOD is one in which the Niño3.4 in the same year does not exceed 1 standard deviation.

## Results

### Seasonality, amplitude, and frequency

**Figure 1:**
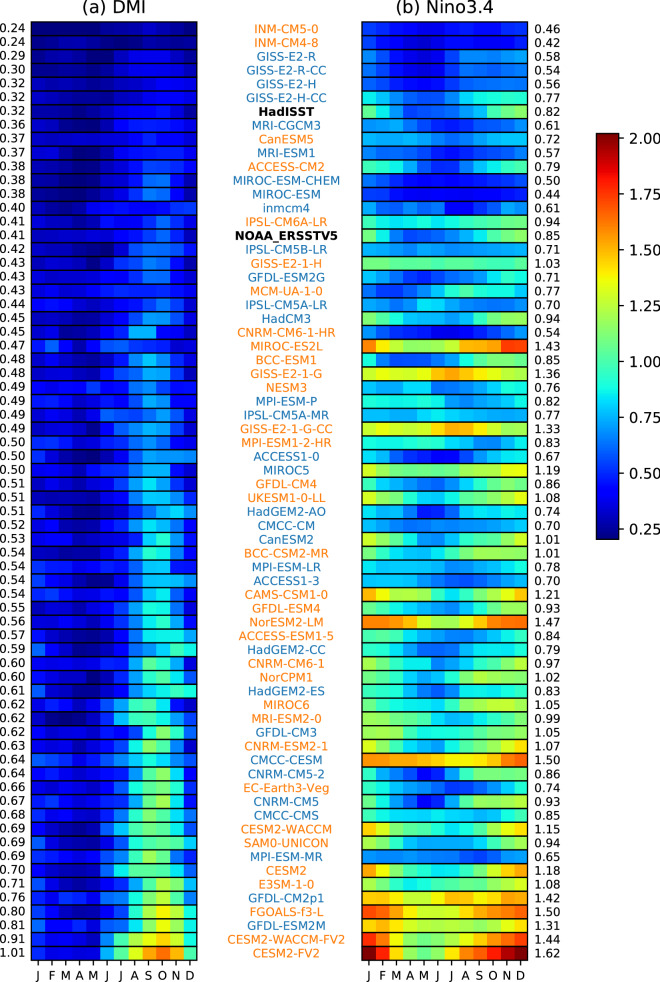
Standard deviation by month for each model in CMIP5 (blue) and CMIP6 (orange) for (**a**) DMI, and (**b**) Niño3.4, an index for IOD and ENSO, respectively during 1850–2005 for models and during 1951–2018 for observations. Both are sorted by the strength of the IOD in ascending order, with strength represented as standard deviation of the index. HadISST and NOAA ERSSTv5 observations shown in bold, to help show models which underestimate and overestimate the IOD.

**Figure 2:**
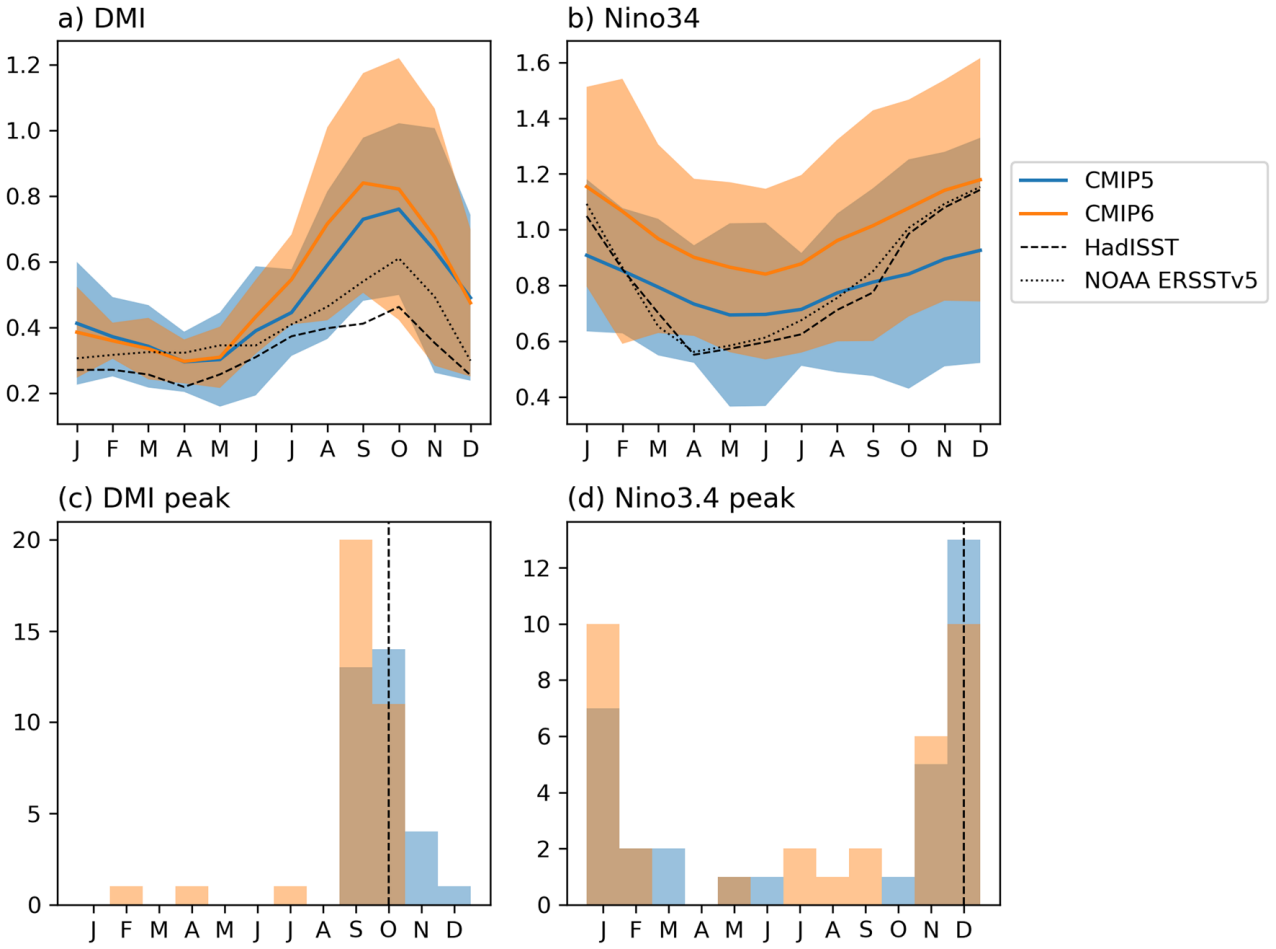
Multimodel mean of monthly standard deviation with interquartile range shaded for CMIP5 (blue) and CMIP6 (orange) in (**a**) DMI, and (**b**) Niño3.4. Peak month histogram of standard deviation for (c) DMI, and (d) Niño3.4, in CMIP5 (blue) and CMIP6 (orange), observed peak in dashed line. Overlap of CMIP5 and CMIP6 is shown in dark orange/brown.

We first examine the strength and seasonality of IOD and ENSO in both CMIP5 and CMIP6. Both IOD and ENSO tend to be phase-locked to certain seasons in the observational record. The IOD begins its development around May–June, peaks in SON, and decays quickly thereafter, while ENSO peaks around DJF. CMIP5 and CMIP6 show relative skill in simulating this seasonal phase locking as exhibited in Fig. 1a,b which shows the standard deviation of DMI and Niño 3.4 for each month. The peak of the IOD and ENSO in CMIP5 and CMIP6 models are identified and shown in a histogram of peak months (Fig. 2c,d). Most CMIP5 models (31/32) correctly simulate the seasonality and peak of the IOD, with only one model peaking outside of SON. In CMIP6, IOD peaks earlier in more models, e.g., no models show a November peak that is otherwise seen in a number of CMIP5 models. Three CMIP6 models peak far too early—in February, April, and July. In contrast to the IOD, the seasonality of ENSO is generally more poorly represented, with many models unable to simulate the observational peak in DJF (Figs. 1 and 2d). It has previously been shown that about a third of CMIP5 models exhibit ENSO mature phase outside the November-to-January peak season in CMIP5^37^. This has not substantially improved in CMIP6 with a similar number of models peaking outside boreal winter.

We also see that in CMIP5 and CMIP6, the IOD is overly strong, with only four out of 32 CMIP5 models and two out of 34 CMIP6 models weaker than the observational data. The bias in overly strong amplitude of IOD does not change in CMIP6, with a relatively similar distribution of IOD strength between CMIP5 and CMIP6. The multimodel mean of the monthly standard deviation for both CMIP5 and CMIP6 for the IOD (Fig. 2a) shows that the magnitude increases in CMIP6, with the interquartile range reduced in non-active IOD months (January–June), and a higher upper quartile range from July onwards. A Student’s t-test suggests that July and August are significantly stronger in CMIP6 compared with CMIP5 at 95% confidence level (*p* < 0.05). The multimodel mean shows that IOD in CMIP6 is still persistently too strong compared to observational products.

Unlike the IOD, ENSO in the CMIP models are not systematically too strong, although there are large differences in their ENSO amplitude. In CMIP6, the amplitude of ENSO appears to have increased overall, with many models showing higher amplitude than in CMIP5. The multimodel mean of monthly standard deviation shows ENSO has increased in strength from CMIP5 to CMIP6 (Fig. 2b). The multimodel mean of monthly standard deviation is much closer to observations in November to January (NDJ), while the phase transition months (March to May and June-July) are too strong (Fig. 2b,d). This suggests model biases in phase locking and ENSO decay^37^. A Student’s t-test has shown that every month is significantly stronger at 95% confidence (*p* < 0.05) in CMIP6 compared with CMIP5, showing a shift in the strength of ENSO. Figure 1a,b also reveals that the strength of the IOD tends to be proportional to the strength of ENSO, with the order of the plot showing the weakest IOD at the top and strongest at the bottom, with ENSO strength ordered by this.

**Figure 3:**
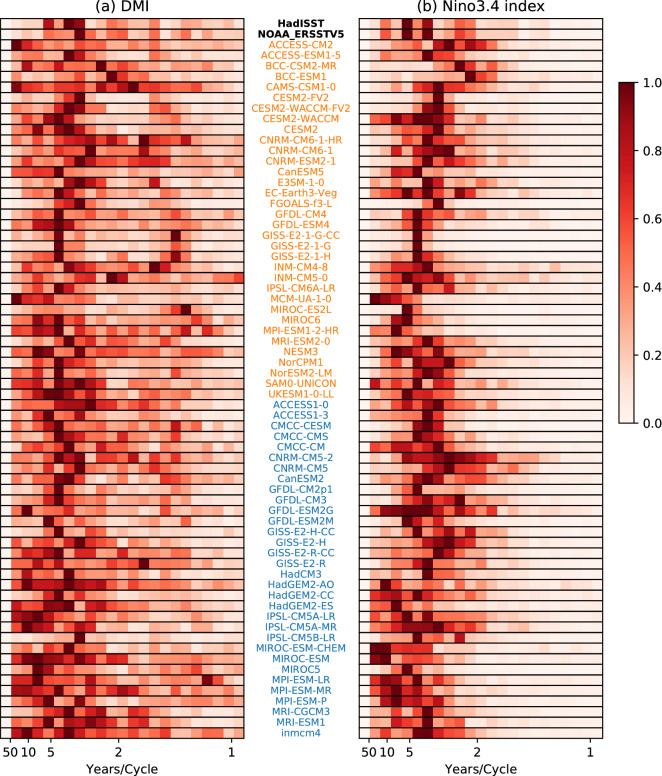
Power spectrum of (**a**) DMI, and (**b**) Niño3.4 index, calculated by Welch's method. Power spectrum of each model is standardised to facilitate inter-model comparison of the ENSO and IOD period. Observations are shown in bold, CMIP5 models in blue text, and CMIP6 models in orange text.

Power spectrum analysis (Fig. 3) reveals how well models simulate the period of IOD and ENSO, and any changes from CMIP5 to CMIP6. In CMIP5, many models (27/32) show the peak power of DMI at higher periods than in observations (Fig. 3a; 5–10 yr/cycle vs 3–5 yr/cycle), although a few models have lower periods (1–2 yr/cycle), e.g., GISS-E2-R, and MPI-ESM-LR. In CMIP6, 20/34 models exhibit peak power at higher period than in observations; i.e., relatively less models than in CMIP5. The reason for this change in periodicity is unclear. Overall there are more models in CMIP6 simulating the period of the IOD better than in CMIP5 (Fig. 3a). For ENSO, the observed peak power of the Niño3.4 index occurs at 2–7 yr/cycle with dominant peaks at 4 and 7 yr/cycle (Fig. 3b). In the models, most see the dominant time scales in the same observed 2–7 yr/cycle range; however, some models see strong variability at higher periods (Fig. 3b). The maximum power is at lower periods for 23/32 CMIP5 and 27/34 CMIP6 models, a significant fraction of both (95% confidence interval, binomial test). From CMIP5 to CMIP6, there has been an overall reduction in the inter-model variability of period, with much of the power limited to the 2–7 year range. This suggests that, compared with CMIP5, CMIP6 models have somewhat improved their representation of both IOD and ENSO periods. However, among CMIP6 models, some now exhibit very narrow period peaks suggesting overly regular ENSO and IOD. For instance, the CMIP6 GISS models exhibit a well-defined peak power of Niño3.4 at about 4 yr/cycle, a period which is also clearly seen in the DMI. Such a feature is less apparent in the CMIP5 versions, thus potentially indicating some systematic changes going from CMIP5 to CMIP6 in the simulation of ENSO which impacts on the IOD reflecting a stronger link between the two phenomena in the CMIP6 version.

**Figure 4:**
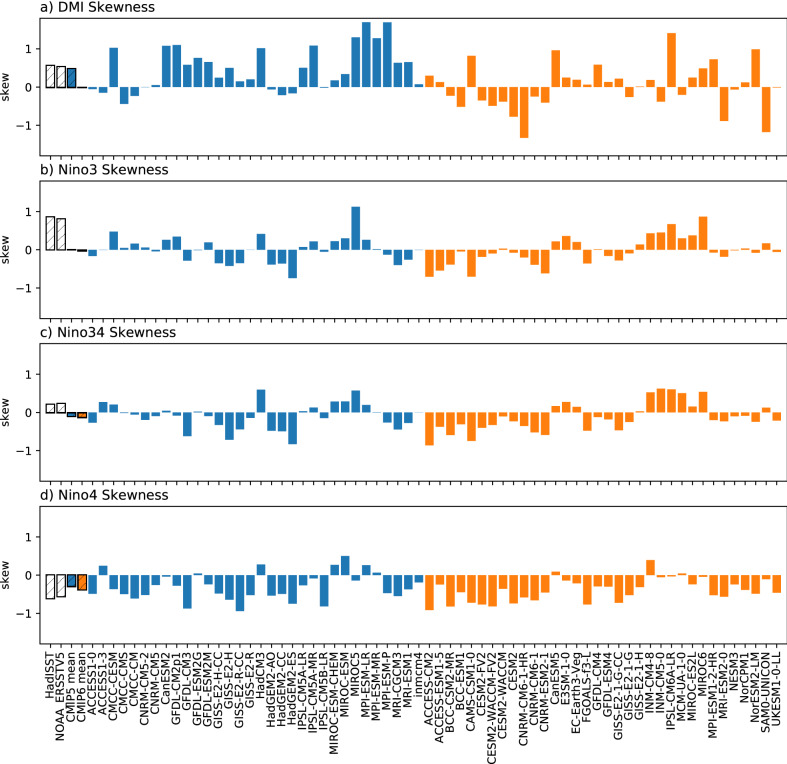
Bar chart showing skewness in (**a**) DMI, (**b**) Niño3, (**c**) Niño3.4 and (**d**) Niño4 for observations (striped), multimodel mean CMIP5 (blue striped), multimodel mean CMIP6 (orange striped), individual CMIP5 (blue) and CMIP6 (orange) models.

We also examine the skewness of the DMI and ENSO indexes, which is a measure of ENSO and IOD asymmetry^5,41,42^. In observations, the DMI and Niño3 show strong positive skewness, indicating that positive events (pIOD or El Niño) tend to be stronger than negative events. The Niño4 index being negatively skewed in observations reflects stronger cool SST anomalies than positive anomalies, associated with the fact that Central Pacific La Niñas tend to be stronger than Central Pacific El Niños. Given the Niño3.4 is located between Niño3 and Niño4, the skewness is weakly positive. For the IOD in CMIP5, most models exhibit positive skewness with varying strength; only a few show very weak negative skewness, resulting in multimodel mean that is close to observations (Fig. 4a). In CMIP6 however there is no systematic skewness across the models, with nearly half of CMIP6 models showing negative IOD skewness. This implies that many CMIP6 models simulate stronger nIOD relative to pIOD, in contrast to observations. For ENSO, there is a large inter-model spread in skewness in both CMIP5 or CMIP6 for Niño3 (Fig. 4b), many showing negative skewness that is at odds with the observations. The Niño3.4 index has shown no improvement from CMIP5 to CMIP6, and continues to represent negative skewness in the majority of models, contrary to observations (Fig. 4c). On the other hand, for the Niño4 index almost all CMIP5 and CMIP6 models have a negative skewness in line with observations, with a slight increase in CMIP6 (Fig. 4d). The most apparent change from CMIP5 to CMIP6 in terms of skewness is that of the IOD.

### Co-occurrences of the IOD and ENSO

**Figure 5:**
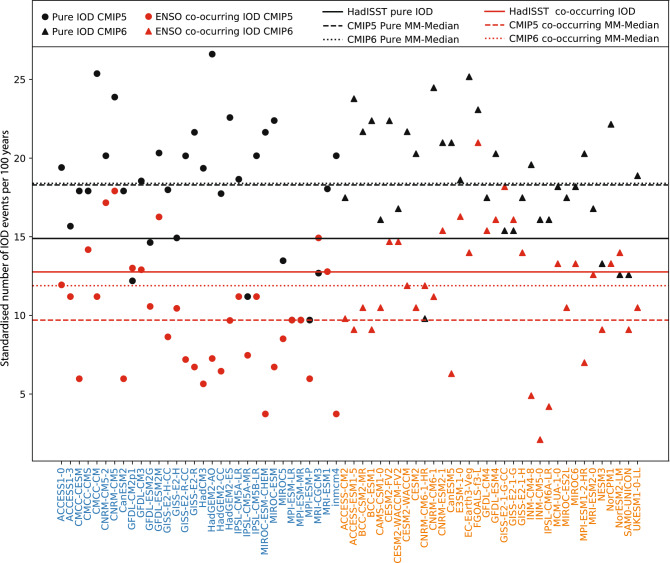
Number of IOD events occurring in each model, standardised to events per century (observed values based on 1950–2018 period also expressed as events per century). IOD events have been separated into pure (no ENSO in the following season), and co-occurring (ENSO occurs in the season following an IOD peak). CMIP5 values are shown in circles, CMIP6 in triangles, with model names ordered alphabetically for CMIP5 first then CMIP6.

As noted in introduction, IOD events can be triggered by ENSO or by dynamics internal to the IO. We examine how the changes in IOD and ENSO frequencies from CMIP5 to CMIP6 are related to changes in the number of co-occurring and independent IOD. Because the observational length is shorter than the models, the number of events for models and observation is standardised in terms of number of events per 100 years. The observational record since 1950 shows that the number of pure IOD events slightly exceeds the number of co-occurring events (15 versus 13 per 100 years, respectively). While this asymmetry is based on a relatively small number of years we find a similar pattern in the models although the difference is much greater in CMIP5; the multimodel median showing 18 independent events versus 10 co-occurring events (Fig. 5). CMIP6 shows a similar result, with the multi model median at around 18 for pure, and 12 for co-occurring IODs. Although the number of pure events is virtually indistinguishable between CMIP5 and CMIP6, there are slightly more co-occurring events in CMIP6 compared with CMIP5 at 90% confidence level. Of note is the higher inter-model variability in the number of co-occurring events among CMIP6, ranging from 2 to 21 events. In terms of the frequency of the overall IOD events, there is a statistically significant increase (90% confidence level) in multimodel median of 30.6 events per 100 years in CMIP5 to 32.2 per 100 years in CMIP6. While the total number of ENSO events also increases slightly from 28.7 (CMIP5) to 31.5 (CMIP6) events per 100 years in the multimodel median, this change is not statistically significant.

Figure 5 shows that there is a significant majority of CMIP5 (25/32) and CMIP6 (30/34) models simulating a higher number of ‘pure’ (i.e., ENSO independent) IODs than observations (significant at 95% confidence level based on binomial test). In CMIP5 a significant majority of models simulate fewer ENSO co-occurring IODs than observations (23/32) (significant at 95% confidence level). This has increased slightly in CMIP6 as mentioned above, with multimodel median higher than in CMIP5. The increase in the number of ENSO co-occurring IODs from CMIP5 to CMIP6 shows a higher association to ENSO events, which may be a result of more ENSO triggered IOD events in CMIP6. Disagreement between models and observations may be due to the different time periods between observation and model; in particular, the observed ENSO and IOD co-occurrences increased toward the late twentieth century^43^. However, inter-model variation tends to be larger than observed trends, and thus the cause for disagreement may boil down to model biases. For instance, contrary to CMIP5, the number of models showing fewer co-occurring events than in observation is in fact not statistically significant in CMIP6.

In our analysis of pure and ENSO co-occurring IOD events, we found no evidence that co-occurrence with ENSO consistently enhances IOD strength in both CMIP5 and CMIP6, underscoring the role of internal processes within the Indian Ocean in IOD growth. In CMIP5 only 4/32 models simulated pIOD and 8/32 simulated nIOD significantly stronger when co-occurring with ENSO versus pure IOD. Only two of these models simulate stronger IOD in both positive and negative co-occurring IODs—MRI-CGCM3, and GFDL-ESM2M. Interestingly the GFDL-CM2.1 model does not have any significant increase in IOD magnitude when IOD and ENSO co-occur, contrary to previous work^24^. This is possibly due to different approaches, with other work using a model experiment forced with observations in the tropical Pacific instead of CMIP historical simulations Pacific Ocean^24^. In CMIP6, a similar number of models showed a significant change when co-occurring: 4/34 for pIOD and El Niño, and 3/34 for nIOD and La Niña. In light of a previous suggestion that an extreme El Niño could be a result of ENSO-IOD interaction^44^ , our analysis found no concrete evidence in CMIP5 and CMIP6 that ENSO is enhanced by IOD events (the magnitude of ENSO events with and without co-occurring IOD events are not significantly different; figure not shown).

**Figure 6:**
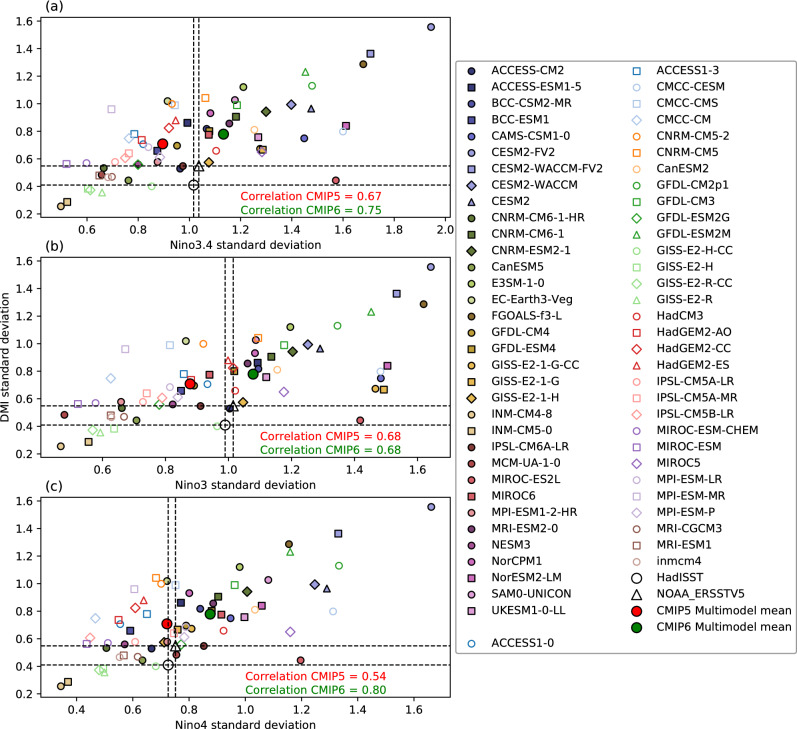
Scatter plots of ENSO strength and IOD strength for different regions of ENSO variability. (**a**) Niño3.4 DJF standard deviation versus DMI SON standard deviation, (**b**) Niño3 DJF standard deviation versus DMI SON standard deviation, (**c**) Niño4 DJF standard deviation versus DMI SON standard deviation. CMIP5 models are indicated with open markers, CMIP6 with filled markers. Correlation coefficients for CMIP5 (red) and CMIP6 (green) are shown in each panel.

Despite that IOD events are not necessarily stronger when co-occurring with ENSO within a model, there is a positive relationship between IOD strength and ENSO strength across models (Fig. 1), although some models with strong IOD can simulate weak ENSO. This relationship is quantified in Fig. 6 that shows the strengths of ENSO versus IOD for each model. Motivated by the observed linkage between IOD strength and ENSO diversity; specifically the link with the strength of cold tongue El Niño and the location of warm pool El Niño (Modoki)^45^, we determine if variability in different parts of the equatorial Pacific are more important in the ENSO-IOD relationship. For this purpose, we use three ENSO indexes that represent variability in the central-to-eastern (Niño3.4), eastern (Niño3), and central (Niño4) equatorial Pacific (Fig. 6a–c, respectively). To understand differences in the relationship from older to newer version of climate model generations, we also calculated correlation and significance for both CMIP5 and CMIP6 separately. From CMIP5 to CMIP6, there is a change in the relationship of ENSO to the IOD. In CMIP5, eastern Pacific ENSO has a higher correlation than central Pacific ENSO with the IOD (Niño3 to DMI correlation = 0.68, Niño4 to DMI correlation = 0.54). However, in CMIP6, the relationship to the eastern Pacific is largely unchanged, but the western Pacific shows a much stronger relationship to the IOD (Niño3 to DMI correlation = 0.68, Niño4 to DMI correlation = 0.80). Thus, western Pacific variability has become a much more prominent determinant of IOD strength in CMIP6. This suggests that some processes have changed between CMIP5 and CMIP6, possibly making a stronger link between warm pool ENSO (or El Niño Modoki) and the Indian Ocean variability. In addition to a shift in regional ENSO variability, we find that the strength of ENSO is increased in the CMIP6 multimodel mean, while the strength of the IOD remains largely similar between CMIP5 and CMIP6. In Fig. 6, we note that the multimodel mean of ENSO strength is larger in CMIP6 than CMIP5 in all measures, as shown by the green dot showing higher magnitude than the red dot. The increase in strength may be an important factor in the enhanced relationship between ENSO and IOD in CMIP6.

### Impact of mean-state bias

**Figure 7:**
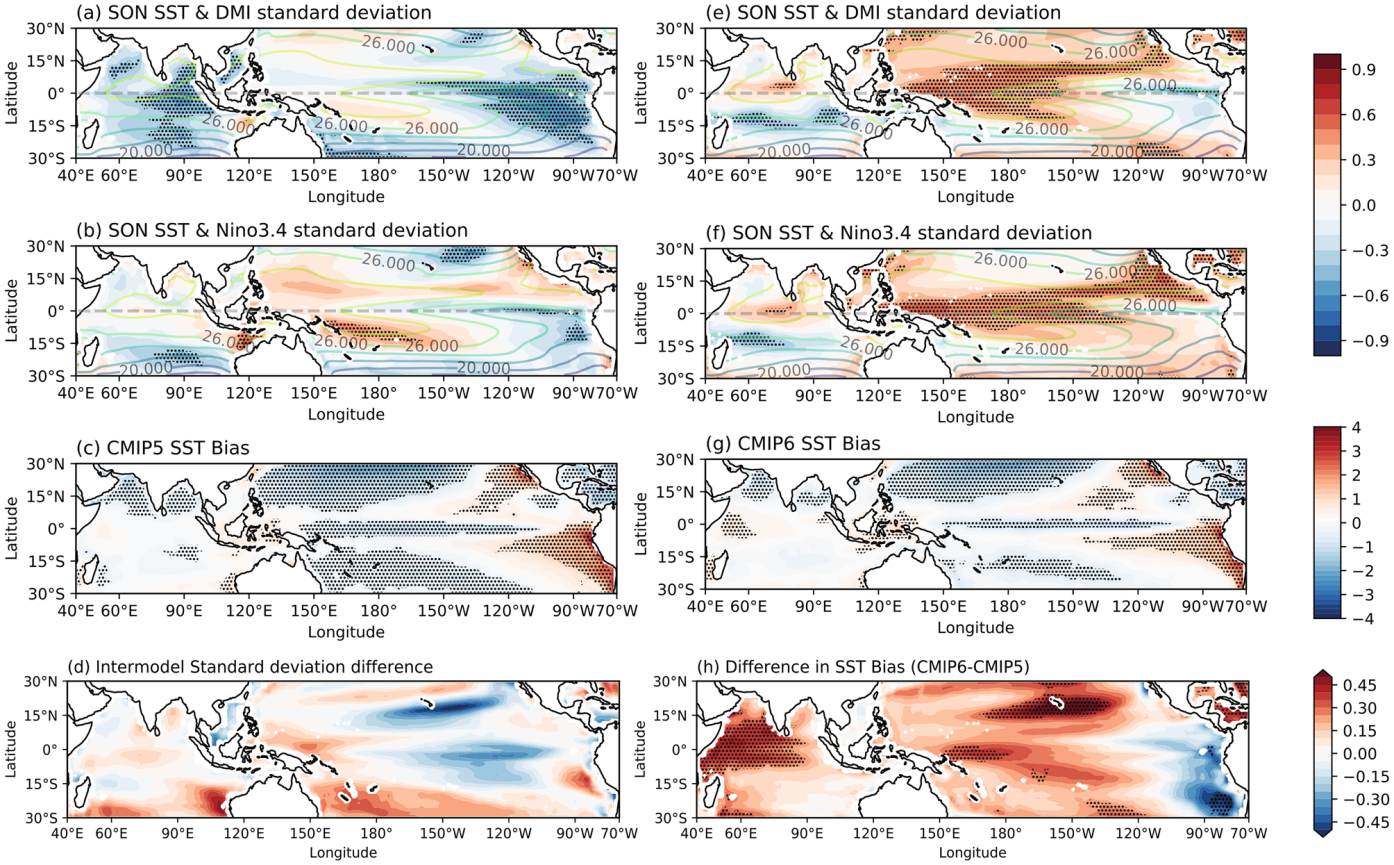
Inter-model correlation of climatological SON average SST to IOD strength (calculated as SON DMI standard deviation) in (**a**) CMIP5, (**e**) CMIP6, and ENSO strength (calculated as DJF Niño3.4 standard deviation) in (**b**) CMIP5 and (**f**) CMIP6. Significant correlations (*p* < 0.05) are stippled. Climatological SON mean SST overlaid in contours, interval = 2 °C. Mean SST bias relative to HadISST (1950–2005) for (**c**) CMIP5, and (**g**) CMIP6 (stippled where differences are significant at a 95% level binomial test). The difference in inter-model standard deviation of SST climatology between CMIP6 and CMIP5 is shown in (**d**). The difference between climatological SST bias is shown in (**h**); stippled where CMIP5 and CMIP6 SST climatology are significantly different at a 90% level based on a Student’s t-test.

Mean state biases have the potential to affect local variability and teleconnections associated with remote variability^28,31^. To this end we examine the link between mean SST bias and the strength of IOD and ENSO in CMIP5 and CMIP6. For this purpose, we calculated an inter-model correlation between mean SON SST at each grid point and IOD strength (Fig. 7a,d), as well as between SON SST and the strength of ENSO (Fig. 7b,f). The inter-model correlation is calculated by first computing the climatological SST in SON at each grid point and the standard deviation of the SON DMI (DJF Niño3.4 index) in each model, and then correlating these two quantities across models. In Fig. 7a,b,e,f, regions of negative (positive) correlations indicate that models with cooler (warmer) sea surface in these regions tend to have stronger IODs. In both CMIP5 and CMIP6, the east Indian Ocean has significant areas of negative correlation (Fig. 7a,e) indicating that models with lower SSTs in this region tend to have stronger IODs. However, in CMIP6, this area is much smaller and located in the southern Indian Ocean around 15°S. The underlying cause for this is not immediately clear and needs to be investigated further in future studies.

Between CMIP5 and CMIP6 the impact of SST bias is considerably different in the Pacific Ocean. In CMIP5, there are large areas of significant negative correlations in the eastern equatorial-south tropical Pacific (Fig. 7a). This implies that models with strong pIOD are associated with cold bias in this eastern Pacific region, which is in turn linked to stronger ENSO, although the region of negative correlation for ENSO is more limited (Fig. 7b). This relationship changes in CMIP6, with the western Pacific Ocean becoming the more important region of influence on IOD strength. Strong positive correlations to both IOD strength and ENSO strength in the western Pacific Ocean, off the Maritime Continent, indicate any biases in the warm pool potentially influencing the strength of both IOD and ENSO in CMIP6 (Fig. 7e,f). Models with warmer SST in the western Pacific tend to have stronger IOD and ENSO. This may also partially explain the higher correlation with the Niño4 index in CMIP6 compared with CMIP5 (Fig. 6c). This is a significant change from CMIP5, which appears to correspond with a systematic change in SST bias throughout models in CMIP6.

While the patterns of SST biases between CMIP6 and CMIP5 are similar (Fig. 7c,g), the difference (CMIP6 minus CMIP5) shows a reduction in the extent of the equatorial Pacific cold tongue bias, with the western equatorial Pacific now warmer in CMIP6 compared to CMIP5 (Fig. 7h). Albeit the limited areas of statistical significance, the overall pattern of this CMIP6-CMIP5 difference in mean SST is to a certain extent analogous to the inter-model correlation pattern between the mean SST and IOD or ENSO strength in CMIP6 (Fig. 7e,f). This highlights the role of mean-state bias which is further exemplified by a statistically significant inter-model correlation between the east-minus-west gradient in mean equatorial Pacific SST and IOD strength (correlation coefficient of -0.60 in CMIP5, -0.46 in CMIP6). Another contributing factor for the change in the inter-model correlation pattern would be the difference in the inter-model spread in SST climatology between the two CMIPs (Fig. 7d). While there is no apparent link with the inter-model correlation pattern, Fig. 7d reveals a smaller inter-model spread in the central-eastern equatorial Pacific in CMIP6. Along with the reduction of cold tongue bias, this could indicate systematic change to processes in the Pacific in CMIP6 which may also be related to changes in mean-state biases in other basins, for instance the Atlantic which is a known region with severe climatological bias that has an impact on the Pacific^46^.

Taken together, these analyses could imply that certain processes have changed from CMIP5 to CMIP6, leading to a stronger link between the IOD and Western Pacific climate, possibly through changes in the characteristics of Central Pacific ENSO (or El Niño Modoki). Other mean-state biases in precipitation and wind for instance may also be important^30,47^, and changes in the frequency of ENSO and/or IOD can also imprint on the mean state. Identifying the exact causes likely requires targeted model experiments (e.g., application of mean-state or flux corrections within partial coupling experiments).

## Discussion and conclusions

The influence of ENSO on the Indian Ocean has been an active research area for several decades, given ENSO’s significant impact on society and environment. ENSO is able to induce an IOD event which in itself presents great climatic risks over Indian Ocean-rim countries. The IODs can also be internally induced within the Indian Ocean^19,20^. Given the importance on atmosphere–ocean circulations, socio-economic impacts and risks, efforts have been put into model improvements in simulating ENSO, IOD, and their links^48,49^. As CMIP6 model outputs are finally available, it is necessary to investigate how the simulations have changed from the previous generation of models in CMIP5. Here we utilise 32 CMIP5 and 34 CMIP6 historical simulations to examine the fidelity in representing the IOD characteristics and its link with the tropical Pacific. We focus on the changes from CMIP5 to CMIP6 in the simulation of the IOD, relationship to ENSO and mean state biases. From CMIP5 to CMIP6 we show that many aspects of the simulation remain similar, but there are some differences and changes relating to ENSO strength and mean state biases.

First, we analyse the basic characteristics of the IOD and ENSO, finding that the seasonality of the IOD in CMIP6 is to a large extent comparable to CMIP5 which matches better to observations than in the case of ENSO. However, there are now more models showing the IOD peaks before November and in a few models peak even outside of SON. In 20 of 34 CMIP6 models the IOD peaks in September. The seasonality of ENSO has not improved overall, with a similar proportion of models peaking far too early or late. Further study on the physical reasons behind this change would prove interesting. In terms of amplitude, the IOD is still overly strong in CMIP6, similar to CMIP5. On the other hand, ENSO strength is increased in the multimodel mean of CMIP6, suggesting that the influence of ENSO to remote locations is also likely to be stronger. With the increase in ENSO magnitude, the peak of ENSO in DJF is closer to observations in CMIP6 than it was in CMIP5 (Fig. 2b). In terms of periodicity or frequency, the simulation of the IOD appears to improve overall in CMIP6, suggesting that models may resolve the associated processes better. There has been an overall reduction in inter-model spread in frequency. However, some models now exhibit very narrow peaks suggesting overly regular ENSO and IOD. The more regular periodicity of ENSO (noted in narrow power spectra), co-occur with more IOD events than in CMIP5. The increased frequency of ENSO may be triggering more IOD events, increasing the co-occurrences of ENSO and IOD in CMIP6 models. These changes in periodicity could be due to possible change in the speed of Rossby and Kelvin wave propagation which plays a role in the phase transition of IOD events^25^.

We also note that CMIP6 models do not systematically capture the observed positive skewness of IOD, which most CMIP5 models were able to simulate (Fig. 4a). This indicates an important loss of realism in IOD simulation in the subset of new models examined here. Further investigation for the systematic change in skewness is warranted, by examining the air-sea feedbacks that govern the IOD^42^. There has been no improvement in the skewness of ENSO from CMIP5 to CMIP6 in the eastern Pacific—neither generation of models consistently show the observed positive skewness in Niño3 (Fig. 4b). Conversely, almost all models are able to simulate the observed negative skewness in Niño4 (Fig. 4d). This indicates that the underestimated asymmetry and nonlinearity associated with Eastern Pacific ENSO in CMIP5^50^.

The relationship between ENSO strength and IOD strength (a positive correlation) has stayed consistent with previous work^6,51,52^, and gotten somewhat stronger from CMIP5 to CMIP6. This suggests that the processes resolving the interactions may have strengthened, in addition to potential changes in the types of ENSO impacting on the IOD. In CMIP6, ENSO is stronger, in both warm pool (Niño4) and cold tongue (Niño3) regions (Fig. 6). The western equatorial Pacific seems to impact on the IOD much more in CMIP6 than in CMIP5, with higher correlation of Niño4 to DMI in CMIP6. This however suggests a contrast to observations in which the amplitude of cold tongue ENSO tends to be more related to the IOD^45^. Our result indicates that the IOD in CMIP6 may be more sensitive to teleconnection with the western Pacific, possibly due to stronger convection there which may be generated more effectively by warm pool ENSO events. The IOD and central Pacific ENSO relationship is complex, with pIOD and warm pool El Niño (Modoki) observed to co-occur before^21,45^—e.g., in 1994 and perhaps more recently in 2019. The change in the relationship from CMIP5 to CMIP6 identified here could reflect systematic changes associated with ENSO diversity, a topic which warrants further investigation. However, our analysis also highlights the role of processes internal to the Indian Ocean, as we found no significant difference in the strength of IOD events when they co-occur with ENSO. Nonetheless, differences with observations may be due to systematic model biases, which are still exhibited in CMIP6 albeit with slight reduction in the extent in some cases, potentially causing ENSO-independent IODs to be overly strong.

In CMIP5, we find that models with lower SON SST climatology in the east IO tend to have stronger IODs^30^. In CMIP6 the relationship is different; the Indian Ocean region is smaller in extent and is located further south indicating models with lower climatological SST having stronger IOD. We also found that the IOD strength in CMIP5 is related to inter-model differences in SST in the eastern Pacific Ocean: models with lower SST in the eastern Pacific Ocean correspond with stronger IOD strength. The relationship is different in CMIP6; instead, models with higher SST in the western equatorial Pacific correspond with stronger IODs. This could be due to a reduction in the cold tongue bias in CMIP6 (Fig. 7c). The analysis essentially reveals that the inter-model correlation between IOD strength and climatological SST changes in sign and location in CMIP6 compared with CMIP5. Further work is required to determine the physical dynamics behind such systematic changes, for instance whether the link associated with model biases is through the atmospheric bridge or the Indonesian Throughflow^48,49,53,54^.

Understanding how simulations of IOD and ENSO progress through climate model generations is important toward more reliable climate predictions and future projections. Past research based on the previous generation of climate models has found that under warming scenarios, the frequency of extreme ENSO events is projected to increase^55–58^. Frequency of extreme pIOD is also projected to increase in future warming scenarios^59^. However, model biases undermine these results, and as the nature of model biases has changed in CMIP6, results from future projection studies need to be re-assessed using CMIP6 models. By the same token, it would be interesting to see how these changes impact on seasonal forecast of ENSO, IOD, and their teleconnection.

## Supplementary information

Supplementary Information.
