# Supplementary material for: Indian Ocean Dipole in CMIP5 and CMIP6: characteristics, biases, and links to ENSO
Source: Sci Rep. 2020 Jul 13;10:11500. doi: 10.1038/s41598-020-68268-9 (PMC7359035; doi:10.1038/s41598-020-68268-9)
Supplement: Supplementary file 1 — Supplementary Information. [file 41598_2020_68268_MOESM1_ESM.pdf]

Indian Ocean Dipole in CMIP5 and CMIP6: Characteristics, biases, and links to ENSO

Sebastian McKenna[1,*], Agus Santoso[1,2,*], Alexander Sen Gupta[1], Andréa S. Taschetto[1], Wenju Cai[2,3]

[1]Australian Research Council (ARC) Centre of Excellence for Climate Extremes and Climate Change Research Centre, The University of New South Wales, Sydney, NSW, Australia
[2]Centre for Southern Hemisphere Oceans Research (CSHOR), CSIRO Oceans and Atmosphere, Hobart, Tasmania, Australia
[3]Key Laboratory of Physical Oceanography/Institute for Advanced Ocean Studies, Ocean University of China and Qingdao National Laboratory for Marine Science and Technology, Qingdao, China
[*] Corresponding Authors
Corresponding emails: s.mckenna@unsw.edu.au; a.santoso@unsw.edu.au

Supplementary Material

Table S1: CMIP5 models and institutions.

Model	*Institute, Country*
ACCESS-1.0	CSIRO-BOM, Australia
ACCESS-1.3	BOM, Australia
CanESM2	CCCMA, Canada
CMCC-CESM	CMCC, Italia
CMCC-CM	CMCC, Italia
CMCC-CMS	CMCC, Italia
CNRM-CM5	CNRM-CERFACS, France
CNRM-CM5-2	CNRM-CERFACS, France
GFDL-CM2p1	NOAA-GFDL, USA
GFDL-CM3	NOAA-GFDL, USA
GFDL-ESM2G	NOAA-GFDL, USA
GFDL-ESM2M	NOAA-GFDL, USA
GISS-E2-H	NASA/GISS, USA
GISS-E2-H-CC	NASA/GISS, USA
GISS-E2-R	NASA/GISS, USA
GISS-E2-R-CC	NASA/GISS, USA
HadCM3	MOHC, UK
HadGEM2-AO	NIMR-KMA,Korea
HadGEM2-CC	MOHC, UK
HadGEM2-ES	MOHC, UK
INMCM4	INM, Russia
IPSL-CM5A-LR	IPSL, France
IPSL-CM5B-LR	IPSL, France
IPSL-CM5A-MR	IPSL, France
MIROC5	AORI-NIES-JAMSTEC, Japan
MIROC-ESM	AORI-NIES-JAMSTEC, Japan
MIROC-ESM-CHEM	AORI-NIES-JAMSTEC, Japan
MPI-ESM-LR	MPI-M, Germany
MPI-ESM-MR	MPI-M, Germany
MPI-ESM-P	MPI-M, Germany
MRI-CGCM3	MRI, Japan
MRI-ESM1	MRI, Japan

Table S2: CMIP6 models and institutions.

Model	*Institute, Country*
ACCESS-CM2	CSIRO-ARCCSS, Australia
ACCESS-ESM1-5	CSIRO, Australia
BCC-CSM2-MR	BCC, China
BCC-ESM1	BCC, China
CAMS-CSM1-0	CAMS, China
CanESM5	CCCma, Canada
CESM2-FV2	NCAR, USA
CESM2	NCAR, USA
CESM2-WACCM-FV2	NCAR, USA
CESM2-WACCM	NCAR, USA
CNRM-CM6-1	CNRM-CERFACS, France
CNRM-CM6-1-HR	CNRM-CERFACS, France
CNRM-ESM2-1	CNRM-CERFACS, France
E3SM-1-0	E3SM-Project
EC-Earth3-Veg	EC-Earth-Consortium
FGOALS-f3-L	CAS, China
GFDL-CM4	NOAA-GFDL, USA
GFDL-ESM4	NOAA-GFDL, USA
GISS-E2-1-G-CC	NASA-GISS, USA
GISS-E2-1-G	NASA-GISS, USA
GISS-E2-1-H	NASA-GISS, USA
INM-CM4-8	INM, Russia
INM-CM5-0	INM, Russia
IPSL-CM6A-LR	IPSL, France
MCM-UA-1-0	UA, USA
MIROC6	MIROC, Japan
MIROC-ES2L	MIROC, Japan
MPI-ESM1-2-HR	MPI-M, Germany
MRI-ESM2-0	MRI, Japan
NESM3	NUIST, China
NorCPM1	NCC, Norway
NorESM2-LM	NCC, Norway
SAM0-UNICON	SNU, Republic of Korea
UKESM1-0-LL	MOHC, UK
